# Enteroenteric Fistula Following Multiple Magnet Ingestion in an Adult: Case Report, Literature Review and Management Algorithm

**DOI:** 10.3390/healthcare13192523

**Published:** 2025-10-05

**Authors:** Laurențiu Augustus Barbu, Liliana Cercelaru, Ionică-Daniel Vîlcea, Valeriu Șurlin, Stelian-Stefaniță Mogoantă, Tiberiu Stefăniță Țenea Cojan, Nicolae-Dragoș Mărgăritescu, Ana-Maria Țenea Cojan, Valentina Căluianu, Mihai Popescu, Gabriel Florin Răzvan Mogoș, Liviu Vasile

**Affiliations:** 1 Department of Surgery, Railway Clinical Hospital Craiova, University of Medicine and Pharmacy of Craiova, 2 Petru Rares Street, 200349 Craiova, Romania; laurentiu.barbu@umfcv.ro (L.A.B.); tiberiu.tenea@umfcv.ro (T.S.Ț.C.); valentina.andronache@yahoo.com (V.C.); 2 Department of Embryology and Anatomy, University of Medicine and Pharmacy of Craiova, 200349 Craiova, Romania; liliana.cercelaru@umfcv.ro; 3 Department of Surgery, Emergency County Hospital, University of Medicine and Pharmacy of Craiova, 2 Petru Rares Street, 200349 Craiova, Romania; ionica.vilcea@umfcv.ro (I.-D.V.); vsurlin@gmail.com (V.Ș.); ssmogo@yahoo.com (S.-S.M.); dmargaritescu@yahoo.com (N.-D.M.); vliviu777@yahoo.com (L.V.); 4 Faculty of Medicine, University of Medicine and Pharmacy of Craiova, 200349 Craiova, Romania; anamariatenea0324@gmail.com; 5 Imaging Department, University of Medicine and Pharmacy of Craiova, 200349 Craiova, Romania; mihai_popescu_rad@yahoo.com

**Keywords:** magnet ingestion, entero-enteric fistula, neodymium, small bowel obstruction, adult, psychiatric disorder, surgery

## Abstract

**Background:** Multiple high-powered magnet ingestion is a surgical emergency due to inter-loop attraction leading to ischemia, necrosis, perforation, and fistula formation. While well documented in children, adult cases—particularly those complicated by entero-enteric fistula—remain rare, and management is largely extrapolated from pediatric guidelines. **Objective:** To present a rare case of adult entero-enteric fistula following multiple neodymium magnet ingestion, we review the literature and propose an adapted management algorithm for adults. **Methods:** A narrative PubMed review was performed to identify pediatric and adult cases of magnet ingestion complicated by gastrointestinal fistula. Search terms included magnet ingestion, entero-enteric fistula, neodymium, and adult. Reported case characteristics, diagnostic modalities, treatments, and outcomes were analyzed. **Results:** A 38-year-old male with schizophrenia presented with small bowel obstruction five days after ingesting multiple magnets. Abdominal radiography revealed clustered radiopaque bodies in the distal ileum. Emergency laparotomy identified an entero-enteric fistula caused by pressure necrosis from inter-loop magnetic attraction. Segmental enterectomy with side-to-side anastomosis was performed, with uneventful recovery. The literature review identified only a few adult cases, which showed similar pathophysiology but frequent diagnostic delays and higher complication rates compared with pediatric cases. **Conclusions:** This case adds to the scarce adult literature on magnet-induced entero-enteric fistula and supports the adaptation of pediatric-based protocols for adults, with attention paid to psychiatric comorbidity and delayed presentation. Early imaging, timely intervention, and multidisciplinary care are essential to prevent severe gastrointestinal injury.

## 1. Introduction

The ingestion of foreign bodies (FBs) is common in children, particularly toddlers and preschoolers, with 5–10% presenting annually to emergency departments (EDs) for suspected ingestion; fewer than 10% require intervention and less than 1% surgery [1]. Coins, batteries, toy parts, and sharp objects are frequent culprits, but the increasing use of small neodymium magnets in toys and decorative kits has created a high-risk category [1,2]. When multiple magnets—or magnets with metallic objects—are ingested, their attraction across bowel walls may create a fixed clamp, leading within days to pressure necrosis, ischemia, perforation, or fistula formation [3,4,5]. Radiographs typically demonstrate clustered or aligned radiopaque bodies that remain stationary despite peristalsis, especially in symptomatic patients [4].

Although this pathophysiology is well established in pediatrics, adult cases are rare and usually associated with psychiatric illness or intentional ingestion. Only isolated reports describe magnet-induced entero-enteric fistulas in adults [6]. We present a rare case of an adult with schizophrenia who developed an entero-enteric fistula after ingesting multiple neodymium magnets, treated by segmental resection with side-to-side anastomosis. To complement this case, we performed a narrative literature review and propose an adapted management algorithm for adults, highlighting the distinctive diagnostic and therapeutic challenges in this population.

## 2. Case Presentation

### 2.1. Patient Information

A 38-year-old Romanian male with schizophrenia on chronic antipsychotic therapy and well-controlled arterial hypertension presented to the County Emergency Hospital Slatina. He was a non-smoker and reported occasional alcohol use. There was no family history of gastrointestinal disease, malignancy, or inherited syndromes. His body mass index (BMI) was 24.3 kg/m^2^, within the normal range. According to the American Society of Anesthesiologists (ASA) classification, he was ASA III owing to psychiatric comorbidity and hypertension. Nutritional assessment revealed mild hypoalbuminemia (Table 1), consistent with protein–energy malnutrition.

### 2.2. Presenting Concerns

Relatives reported the ingestion of multiple small magnets approximately five days before admission. Within 24 h, the patient developed diffuse abdominal pain, followed by progressive distension, repeated episodes of food-containing vomiting, and altered bowel transit, with absence of stool and flatus for the preceding 48 h.

### 2.3. Clinical Findings

On admission, the patient was hemodynamically stable but mildly dehydrated. Vital signs included blood pressure 135/85 mmHg, heart rate 98 bpm, respiratory rate 20/min, temperature 37.8 °C, and oxygen saturation 97% on room air. The qSOFA score was 0. Abdominal examination showed marked distension with diffuse tenderness, without guarding or rebound. Bowel sounds were hyperactive.

### 2.4. Diagnostic Assessment

**Figure 1:**
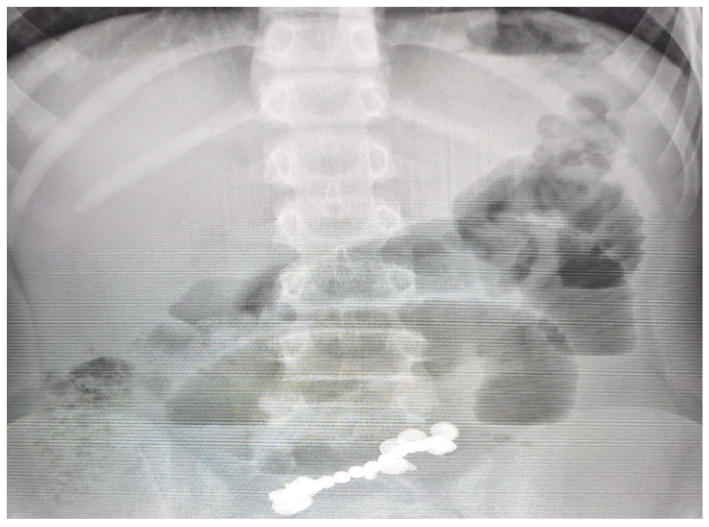
Plain abdominal radiograph demonstrating multiple radiopaque spherical foreign bodies clustered in the distal ileal region, consistent with ingested high-powered magnets. Associated bowel gas pattern suggests small bowel obstruction.

Plain anteroposterior abdominal radiography demonstrated multiple clustered radiopaque foreign bodies within the distal ileal loops, consistent with ingested magnets (Figure 1). No free intraperitoneal air was detected. Although no lateral decubitus or serial radiographs were performed, magnet stationarity was inferred from the clinical course. Laboratory tests showed leukocytosis (WBC 15,000/mm^3^), hyponatremia (132 mmol/L), hypokalemia (3 mmol/L), and hypochloremia (90 mmol/L), attributable to persistent vomiting and gastrointestinal losses. C-reactive protein was elevated (120 mg/L), indicating systemic inflammation, while serum albumin was reduced (2.8 g/dL), consistent with malnutrition and catabolic stress. Preoperative management included intravenous fluid resuscitation, electrolyte correction, nasogastric decompression, and prophylactic broad-spectrum antibiotics. Collectively, these abnormalities, together with the clinical and imaging findings, indicated the need for urgent surgical intervention (Table 1).

### 2.5. Therapeutic Intervention

Given the radiological findings and clinical suspicion of small bowel complication, emergency surgery was indicated. Although minimally invasive access was initially considered, laparoscopic exploration was deemed unsafe due to marked bowel distension and the high risk of fistula or perforation; therefore, a midline laparotomy was performed under general anesthesia.

**Figure 2:**
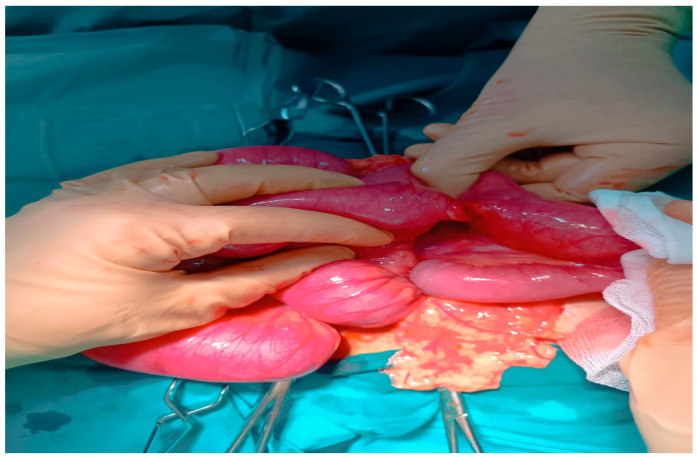
Intraoperative findings during midline laparotomy showing two adjacent small bowel loops adherent to each other, consistent with an entero-enteric fistula secondary to pressure necrosis caused by ingested high-powered magnets. The serosal surfaces appear hyperemic and edematous, with focal transmural involvement at the fistula site. Surrounding mesentery demonstrates inflammatory changes.

**Figure 3:**
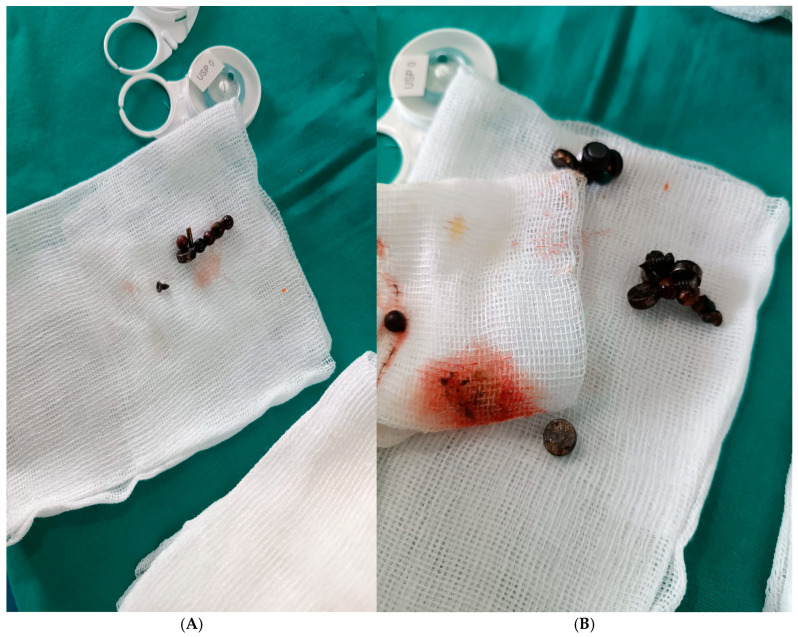
(**A**,**B**): Intraoperative retrieval of multiple ingested high-powered neodymium magnets. The magnets, some spherical and others disk-shaped, were removed en bloc from different small bowel segments following segmental enterectomy for an entero-enteric fistula. Corrosion and surface discoloration are visible, indicating prolonged gastrointestinal exposure. These foreign bodies were identified preoperatively on abdominal radiography as clustered radiopaque densities.

Intraoperative exploration revealed a 10 mm entero-enteric fistula approximately 100 cm distal to the duodenojejunal flexure, caused by transmural pressure necrosis from magnets adhering across adjacent small bowel loops (Figure 2). Thirteen neodymium magnets (9 spherical, 4 disk-shaped) were retrieved en bloc from the resected segment (Figure 3). Additional findings included serosal hyperemia and inter-loop attraction sites, but no perforation. Systematic inspection of the entire small bowel confirmed the absence of subserosal collections or further fistulous tracts. The affected ileal segment (18 cm) was resected, followed by side-to-side anastomosis.

### 2.6. Postoperative Course and Follow-Up

The postoperative course was uneventful. Broad-spectrum intravenous antibiotics (ceftriaxone and metronidazole) were administered for 5 days, together with low-molecular-weight heparin prophylaxis for 7 days. Laboratory monitoring at 24, 48, and 72 h showed progressive normalization of inflammatory markers (CRP: 120 → 75 → 25 mg/L; WBC: 15.0 → 11.0 → 8.5 × 10^9^/L).

Oral feeding was resumed gradually, with clear liquids on postoperative day 2 and a soft diet from day 4. Bowel transit was restored by postoperative day 5. The patient was discharged in good condition on postoperative day 7, with primary wound healing.

Histopathological examination of the resected ileal segment (18 cm) demonstrated transmural pressure necrosis with localized inflammatory infiltration, serosal fibrosis, and viable resection margins.

At 1- and 3-month follow-up, including clinical examination and radiological evaluation, the patient remained asymptomatic with normal bowel function and no evidence of stricture or recurrent fistula. He continues psychiatric supervision to address the underlying behavioral risk and prevent recurrence.

### 2.7. Patient Perspective

The patient expressed relief at the resolution of abdominal symptoms and was cooperative during hospitalization. Family members were informed about the risks of foreign body ingestion and the importance of strict psychiatric supervision.

### 2.8. Informed Consent

Written informed consent was obtained from the patient for the publication of this case report and any accompanying clinical images.

### 2.9. Timeline of Clinical Events

A structured timeline of key clinical events from ingestion to follow-up is presented in Table 2.

### 2.10. Literature Review Methods

A narrative literature review was performed using the PubMed database. The search strategy combined the terms “*magnet ingestion*,” “*neodymium*,” “*entero-enteric fistula*,” “*gastrointestinal fistula*,” and “*adult*” with Boolean operators (AND/OR). The search covered publications from January 1990 to April 2024, with the last update on 15 June 2025.

Eligibility criteria included case reports, case series, observational studies, and review articles reporting gastrointestinal fistulas related to magnet ingestion in pediatric or adult patients. Exclusion criteria were studies not involving fistula formation, experimental or animal research, non-English publications, duplicate records, and articles without accessible full texts.

Two authors (L.A.B. and T.S.Ț.C.) independently screened titles and abstracts, with full texts assessed when eligibility was uncertain. Discrepancies were resolved by consensus.

## 3. Discussion

### 3.1. Epidemiology and Clinical Relevance

Foreign body (FB) ingestion is a frequent cause of emergency department (ED) visits in children under five years of age but also occurs in adults, typically accidentally or in association with psychiatric disorders or incarceration [1,7]. Most FBs pass spontaneously; however, sharp objects, button batteries, and particularly multiple high-powered neodymium magnets carry a high risk of gastrointestinal injury, including perforation and fistula formation [3,4].

Neodymium magnets exert strong mutual attraction, and when located in separate bowel loops, they can entrap intervening intestinal walls (“magnetic clamp” effect), leading to ischemia, pressure necrosis, and transmural injury. These complications may develop rapidly or manifest later as entero-enteric or entero-colonic fistulas [3,6,8].

Following the relaxation of commercial restrictions, pediatric studies have reported a rising incidence of magnet ingestion, with up to one-third of cases requiring surgery and complications ranging from multiple perforations to chronic fistulas with malabsorption [3,9]. Although rare in adults, magnet ingestion poses similar diagnostic and therapeutic challenges, particularly when ingestion is unwitnessed or the clinical history is unclear [4,9]. The present case of adult entero-enteric fistula adds to the limited literature and underscores the need for early diagnosis and multidisciplinary management.

### 3.2. Pediatric vs. Adult Prevalence

The vast majority of magnet ingestions occur in children. In a five-country multicenter study (2011–2021) including 189 pediatric cases, 46.6% involved multiple magnets and 28% required surgery, with significantly higher intervention rates in multiple-magnet ingestions. A prospective Spanish ED network study reported an incidence of 4.8 per 100,000 visits; 31% involved multiple magnets, and 15% required endoscopic extraction overall (36% in multiple ingestions), with no surgeries needed. These findings underscore differences across healthcare systems and the benefits of early access to care [10].

In contrast, adult cases are uncommon but clinically significant, most often associated with intentional ingestion or psychiatric disorders [4]. One report described a 21-year-old male who ingested two magnets, resulting in a jejuno-ileal fistula and small-bowel obstruction necessitating surgical resection [3].

### 3.3. Time to Complications

Multiple neodymium magnets can attract across adjacent bowel loops within 12–48 h, causing pressure necrosis, ischemia, and perforation if not promptly removed [11]. Beyond the acute phase, delayed fistula formation is well documented. A pediatric systematic review of non-acute cases reported a mean interval of 22.8 days (range: 1–90) from ingestion to fistula detection [6], with additional series describing cases after 2, 30, and 90 days of retention [6]. Radiologic warning signs include persistent clustering or fixed positioning of magnets on serial abdominal imaging despite active peristalsis, accompanied by progressive symptoms [11].

### 3.4. Fistula Types Reported

Documented fistula patterns include small bowel–small bowel (ileo-ileal, jejuno-ileal) [12,13,14], gastroduodenal and duodeno-jejunal, some of which were successfully managed endoscopically or conservatively [13,15,16,17], and jejuno-colonic fistulas in delayed pediatric cases, often associated with chronic malabsorption or volvulus [6,18]. Additional reports describe entero-enteric fistulas requiring segmental resection with primary anastomosis [3,19].

### 3.5. Pathophysiological Mechanisms

Ingestion of multiple high-powered magnets carries a distinct risk due to mutual attraction across non-adjacent bowel loops, entrapping the intervening walls and exerting sustained pressure that compromises blood flow, leading to ischemia, necrosis, and transmural injury. This process may progress to perforation or fistula formation when adjacent loops adhere and establish abnormal communications [3,20].

Acute injury can develop within 12–48 h, whereas delayed presentations—ranging from days to months—occur when magnets remain undetected [1,3]. Magnetic forces may counteract peristalsis, producing fixed positions visible radiographically as persistent clustered opacities despite bowel motility [3]. When ingested at different times, magnets may anchor in separate segments (e.g., jejunum and ileum), creating fixed points that obstruct transit, promote mucosal erosion, and result in entero-enteric, gastro-duodenal, or jejuno-colonic fistulas [3,21,22,23,24,25]. In some cases, these fixed points serve as an axis for secondary volvulus, further accelerating ischemia and bowel wall injury [22].

### 3.6. Role of Early Diagnosis and Rapid Intervention

Prompt recognition and early intervention are essential to prevent severe gastrointestinal injury from multiple neodymium magnet ingestion. Attraction across bowel walls can produce mucosal damage within hours and progress to ischemia, necrosis, or fistula formation within days [6]. When ingestion history is unclear or presentation is delayed—particularly in intentional or psychiatric cases—diagnosis becomes more challenging and chronic complications more likely [3,6]. Persistent clustering of magnets on serial imaging despite bowel motility is a key radiologic warning sign for early detection [6].

### 3.7. Comparative Clinical, Diagnostic, and Therapeutic Features in Pediatric Versus Adult Magnet Ingestion

#### 3.7.1. Clinical Presentation

Both pediatric and adult cases typically present with abdominal pain, vomiting, and signs of bowel obstruction [4,6]. In chronic presentations, an initial symptom-free interval may precede progressive gastrointestinal complaints [16,17]. Risk factors differ by age: accidental ingestion predominates in children [18], whereas psychiatric history and intentional ingestion are more common in adults [4]. Pediatric cases are often complicated by delayed disclosure and uncertain timing of ingestion [1], while in adults the history may be clearer but sometimes intentionally concealed, particularly among prisoners or psychiatric patients [4]. Chronic malabsorption and systemic complications such as hepatosteatosis or renal lithiasis are mainly described in delayed pediatric cases [6] and are uncommon in acute adult presentations.

#### 3.7.2. Diagnosis

Plain abdominal radiography is the first-line investigation, with persistent clustering of magnets in the same location on serial films serving as a red flag for inter-loop attraction and potential fistula formation [19,20]. When radiographs are inconclusive, CT or MRI can confirm fistulas and assess complications [3,21]. MRI, however, is generally contraindicated until magnet ingestion has been excluded. Diagnostic delay is strongly associated with fistula development in both children and adults [1,22]. While pediatric protocols may employ MRI or contrast studies (e.g., barium enema) for fistula confirmation [6], CT is the preferred modality in adults, particularly in acute presentations or when the ingestion history is unclear [3,4].

#### 3.7.3. Treatment and Outcomes

Multiple magnet ingestions complicated by fistula formation generally require surgical intervention in both pediatric and adult patients [3,23]. Laparotomy with resection and primary anastomosis is the standard approach for obstruction, perforation, or large fistulas [24,25]. In children, delayed presentation often necessitates extensive resections, multiple anastomoses, or volvulus repair, with long-term sequelae such as malabsorption [6]. In adults, earlier surgery is typically more straightforward, usually limited to segmental resection or fistula closure, with fewer systemic consequences [3,4]. Endoscopic retrieval is feasible only in early cases, predominantly in children, and is rarely an option once obstruction or fistula has developed [1,3].

Prognosis depends on the number of magnets, timing of diagnosis, and feasibility of endoscopic removal. A multicenter pediatric cohort from the Gulf region, North Africa, and Turkey reported markedly higher morbidity in multiple-magnet cases—perforation 44.3%, necrosis 19.3%, peritonitis 13.6%—with surgery required in 59.1% versus 1.0% for single magnets, but no mortality [1]. In contrast, a Spanish ED network study reported favorable outcomes with early presentation and selective endoscopy, achieving 15% overall endoscopic interventions (36% in multiple ingestions) without surgery or complications [10].

Guideline-based management also influences outcomes. NJPIES data (2021–2022) associated expectant management with longer hospital stays; NASPGHAN data indicated 52% required endoscopy alone, 20% combined endoscopy and surgery, 8% surgery alone, and 15% observation, with 41% of surgeries addressing perforation or fistula and 22% involving bowel resection [7,14,24]. In a Chinese single-center series (n = 100), 33% required surgery, which was associated with ingestion of more magnets (median 7.5 vs. 4), longer intervals from ingestion to presentation, and higher rates of perforation or obstruction [9]. Similarly, Tsai et al. reported that 80% of surgical patients had perforation or fistula, all treated with laparoscopic-assisted exploration and a mean postoperative stay of 4 days [2].

Taken together, both pediatric and adult data demonstrate that delayed diagnosis significantly increases the likelihood of bowel resection rather than simple magnet removal.

### 3.8. Psychiatric Implications of Magnet Ingestion in Adults

Although most magnet ingestions occur in children, adult cases—though rare—are typically intentional and associated with psychiatric disorders, intellectual disability, substance abuse, or self-harm [18,25,26,27,28]. Ingestion may be impulsive, repetitive, or concealed, delaying diagnosis and increasing the risk of advanced gastrointestinal injury.

Diagnostic challenges stem from unclear histories, particularly in psychiatric or institutional settings, and often result in complex intraoperative findings such as adhesions, perforations, and fistulas. Pediatric management principles—urgent removal of multiple magnets, avoidance of prolonged observation, serial imaging, and early surgical consultation—are directly applicable to adults [1,4,29]. In acute cases, CT is preferred over MRI due to safety concerns when magnets are retained [3,30].

Prevention should extend beyond pediatric awareness campaigns to include institutional safety protocols in psychiatric wards and correctional facilities, as well as regulatory measures on high-strength neodymium magnet sales. Postoperative care must incorporate structured psychiatric evaluation to address behavioral risks and reduce recurrence, reflecting the multidisciplinary approach also recommended in pediatric practice [18,31,32].

### 3.9. Limitations in Reported Adult Cases

Magnet ingestion leading to entero-enteric fistula is exceedingly rare in adults, with only a few documented cases. In a review of 149 magnet-ingestion injuries over two decades, 6 cases (4%) involved adults, the oldest aged 48 years [18]. This scarcity limits adult-specific evidence, and most clinical knowledge is extrapolated from pediatric reports. Given the risk of multiple magnets attracting across bowel loops and causing necrosis, perforation, or fistula [29], experts recommend early surgical intervention even in asymptomatic cases [30]. In the absence of adult guidelines, management generally follows pediatric protocols.

Diagnostic delays are common, as adults often present without a clear ingestion history, particularly in unwitnessed or accidental cases. Symptoms such as abdominal pain, nausea, or obstruction are non-specific, leading to late recognition [31]. In one case, a patient was unaware of swallowing two magnets, which were missed on radiographs and discovered intraoperatively. A systematic review, predominantly pediatric, reported a mean interval of ~23 days (range 1–90) from ingestion to fistula detection, with nearly all patients requiring surgical repair (47/55 cases).

The combination of vague symptoms and low clinical suspicion frequently postpones intervention, resulting in more extensive surgery. Once multiple magnets pass beyond the stomach, expert consensus supports prompt exploratory surgery to prevent peritonitis, as delayed management markedly increases morbidity.

The true incidence of adult cases is likely underestimated due to reporting bias, as mild or conservatively managed cases are seldom published. Similar patterns are observed in other rare abdominal conditions, including appendiceal malakoplakia (typically reported as isolated case reports) [32], mesenteric cysts (mainly described through single-case publications) [33], and vascular entities such as thrombosed abdominal aortic dissecting aneurysm with ischemic colitis (reported only sporadically) [34]. This selective reporting highlights the need for systematic data collection to more accurately define the incidence and outcomes of adult magnet ingestion.

The main limitation of the proposed algorithm is the low level of evidence, as it is based on a small number of reported adult cases. Larger, prospective studies are required to validate its applicability and outcomes.

### 3.10. Implications for Clinical Practice

Magnet ingestion should be suspected in adults presenting with unexplained acute abdominal symptoms, particularly those with intellectual disabilities, psychiatric disorders, or incarceration history, as these groups are at increased risk of intentional foreign body ingestion [32,33,34,35,36,37]. Even in otherwise healthy adults, unusual metallic densities on imaging warrant consideration of magnets due to their potential for life-threatening gastrointestinal injury.

Abdominal radiography is the first-line diagnostic tool, typically demonstrating radiopaque magnets in a clustered or “chain-of-beads” configuration. If inconclusive, computed tomography should be used for localization and complication assessment. Magnetic resonance imaging must be avoided until ingestion is excluded, as strong magnetic fields may displace retained magnets and exacerbate injury [38].

Management should be risk-stratified. A single magnet beyond the stomach may be monitored with serial radiographs, although endoscopic retrieval is preferred when feasible. In contrast, multiple magnets or a magnet combined with another metallic object constitute high-risk scenarios. Urgent endoscopic removal is indicated if these are located in the stomach or proximal duodenum [5]. Once beyond the stomach, multiple magnets should be assumed to attract across bowel loops, predisposing to ischemia, perforation, fistula, or obstruction [39]. In such cases, early surgical exploration is recommended, as conservative management is consistently associated with poorer outcomes.

The development of adult-specific protocols is warranted. These should emphasize targeted history-taking (with attention to magnet exposure and psychiatric risk factors), routine radiography in suspected cases, early surgical or gastroenterology consultation for confirmed or suspected multiple magnet ingestion, and structured observation protocols with predefined criteria for operative escalation [20]. Safety measures such as mandatory abdominal radiography before MRI in neurologically or cognitively impaired patients should also be incorporated [40].

Adult-oriented guidelines, aligned with pediatric principles, would support timely recognition and intervention. Evidence consistently demonstrates that early removal significantly reduces progression to complex injuries such as entero-enteric fistulas [25,41,42,43]. Heightened clinical suspicion, rapid imaging, and decisive management remain central to improving outcomes in this rare but high-risk condition.

### 3.11. Proposed Adult Management Algorithm for Multiple Magnet Ingestion

Although pediatric guidelines for multiple magnet ingestion are well established, adult-specific protocols remain absent due to the rarity of reported cases. Consequently, most adult management strategies are adapted from pediatric algorithms, with modifications for intentional ingestion, psychiatric comorbidity, and the higher likelihood of delayed diagnosis. The following framework outlines an evidence-informed approach for adults, integrating principles from pediatric guidelines (NASPGHAN/ESPGHAN) with insights from adult case reports and series.

**Step 1** **—Initial Assessment**

A detailed history should be obtained, including ingestion timing and the possibility of sequential ingestion, followed by thorough clinical examination. Anteroposterior and lateral abdominal radiographs are first-line investigations to determine the number and location of magnets. Computed tomography is indicated when localization is uncertain or complications are suspected [4,41,42,43].

**Step 2** **—Risk Stratification**

- *Single magnet, asymptomatic*: observation with serial radiographs ≤ 24 h to confirm progression.
- *≥2 magnets or magnet + metallic object*: high risk for ischemia and fistula formation, warranting expedited intervention [4,28].

**Step 3** **—Intervention**

- Magnets in stomach or accessible duodenum: urgent endoscopic removal within 12 h.
- Distal magnets, asymptomatic, with documented progression: inpatient observation, nil per os, clinical monitoring, and abdominal radiographs every 8–12 h; surgical exploration is indicated for peritonitis signs or lack of progression [28].
- Distal magnets with symptoms, stagnation, or perforation signs: laparotomy or laparoscopy with resection if required [4].

**Step 4** **—Post-Intervention**

- All intentional ingestion cases should undergo postoperative monitoring and psychiatric evaluation.

This adapted algorithm emphasizes the importance of rapid diagnosis, timely intervention, and multidisciplinary management in adult multiple magnet ingestion. While the pathophysiological risks mirror those in children, adults often present with additional diagnostic and psychosocial challenges, particularly in the context of psychiatric illness or intentional ingestion (Table 3).

Compared with pediatric guidelines (NASPGHAN/ESPGHAN), the proposed algorithm for adults incorporates three major differences. First, clinical presentation in adults is often delayed, which requires early use of computed tomography for localization and complication assessment, whereas in children serial radiographs are usually sufficient. Second, in the adult population intentional ingestion predominates, particularly in patients with psychiatric disorders or those in institutionalized settings; therefore, our algorithm includes mandatory post-intervention psychiatric evaluation to prevent recurrence. Third, the threshold for surgical exploration is lower in adults, especially in cases of poor compliance, suspected repeated ingestions, or radiologic stagnation beyond 24 h, unlike in pediatrics, where endoscopy and close monitoring remain the priority. Thus, the proposed algorithm adapts pediatric principles to the diagnostic and psychosocial particularities of the adult patient.

Until dedicated adult guidelines are available, pediatric-based principles remain the cornerstone of management, supplemented by early psychiatric assessment, a lower threshold for surgical exploration, and structured inpatient monitoring. This approach aims to prevent severe complications such as perforation, volvulus, and entero-enteric fistula. It must be emphasized, however, that the proposed adult algorithm is extrapolated from pediatric guidelines and limited adult case reports, and has not been validated in prospective studies; therefore, it should be applied with caution until further evidence emerges.

**Figure 4:**
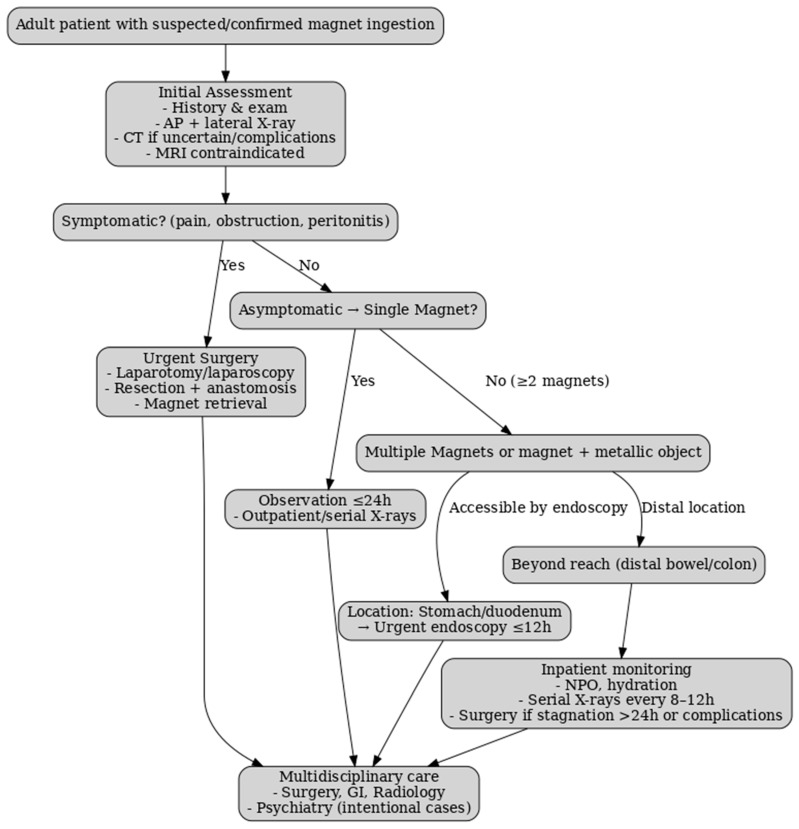
Decision tree for adult management of multiple magnet ingestion. The decision tree integrates pediatric-based principles with adult-specific considerations. It incorporates clear timeframes (≤12 h for urgent endoscopy, 8–12 h intervals for serial X-rays, ≤24 h stagnation before surgery), highlights the role of CT when history is uncertain, and specifies MRI contraindication. Adapted from NASPGHAN/ESPGHAN pediatric guidelines, adult case reports, and adult foreign body guidelines (ESGE, ASGE) [44,45].

To operationalize this strategy, the framework was converted into a decision tree incorporating explicit timeframes (≤12 h for urgent endoscopy, 8–12 h for serial radiographs, ≤24 h stagnation before surgery), defined surgical triggers, and clear imaging recommendations (CT versus radiography, with MRI contraindicated). This evidence-informed decision tree is presented in Figure 4.

### 3.12. Comparison with Reported Cases in the Literature

To contextualize the present case and assess similarities and differences in presentation, mechanism, and management, a focused literature review was conducted on reports of magnet ingestion complicated by gastrointestinal fistula (Table 4). The collected cases, predominantly pediatric with rare adult presentations, allow direct comparison with our findings and highlight diagnostic and therapeutic patterns.

Of the 13 reported cases, 84.6% occurred in children and 15.4% in adults. Fistulas were identified in 61.5% of patients, with surgical intervention required in 61.5%, endoscopic removal in 30.8%, and multidisciplinary management in 7.7%. Pediatric cases predominated and frequently necessitated bowel resection, while adult cases, though fewer, demonstrated similar mechanisms. The high rate of surgery underscores the importance of early detection, which increases the likelihood of successful endoscopic management before fistula formation.

Psychiatric comorbidity is a critical determinant in adult magnet ingestion, contributing to delayed diagnosis and recurrence risk. Surgeons should maintain heightened suspicion in psychiatric patients presenting with unexplained abdominal symptoms, while structured psychiatric evaluation and follow-up are essential to prevent recurrence.

The present case represents one of the few documented instances of entero-enteric fistula secondary to multiple magnet ingestion in an adult. It contributes to the limited literature by outlining the diagnostic challenges, operative findings, and applicability of pediatric-based management principles in an adult psychiatric context. It further emphasizes the need for heightened clinical suspicion, early imaging, and multidisciplinary involvement to optimize outcomes in this rare but high-risk condition.

## 4. Conclusions

Entero-enteric fistula following multiple magnet ingestion is exceptionally rare in adults, with only a few cases reported. This case highlights the distinctive challenges of delayed presentation in psychiatric patients and provides adult-specific insights that are largely absent from the literature. Early recognition, timely imaging, and multidisciplinary intervention remain essential to prevent severe complications. Until dedicated adult guidelines are developed, adapting pediatric algorithms—while integrating systematic psychiatric assessment and maintaining a lower threshold for surgery—offers the most pragmatic strategy. The originality of the proposed algorithm lies in adapting pediatric principles to the adult context, by emphasizing the risk of delayed diagnosis, the increased frequency of intentional ingestions, and the need for systematic psychiatric evaluation, together with a lower surgical threshold to prevent severe complications. Nevertheless, the level of evidence supporting this algorithm remains limited, and larger prospective studies are required to validate its applicability.

## Figures and Tables

**Table 1:** Laboratory findings at admission (SI units).

Parameter	Result (SI Units)	Reference Range (SI Units)
**WBC**	**15.0 ×** **10^9^/L**	4.0–10.0 × 10^9^/L
**Sodium (Na^+^)**	**132 mmol/L**	135–145 mmol/L
**Potassium (K^+^)**	**3.0 mmol/L**	3.5–5.0 mmol/L
**Chloride (Cl^−^)**	**90 mmol/L**	98–106 mmol/L
**CRP**	**120 mg/L**	<5 mg/L
**Albumin**	**28 g/L**	35–50 g/L

WBC = white blood cells; CRP = C-reactive protein.

**Table 2:** Timeline of clinical events, interventions, and follow-up in a 38-year-old patient with magnet-induced entero-enteric fistula.

Day/Time	Event
**Day 0**	Ingestion of 13 neodymium magnets (9 spheres, 4 disks)
**Day 5**	Admission to emergency department with abdominal pain, distension, vomiting; ASA III, hemodynamically stable (BP 135/85 mmHg, HR 98 bpm, Temp 37.8 °C, SatO_2_ 97%); mild dehydration noted
**Day 5**	Abdominal X-ray: clustered radiopaque bodies in distal ileum; no free air
**Day 5**	Preoperative management: IV fluid resuscitation, electrolyte correction, nasogastric decompression, prophylactic antibiotics (ceftriaxone + metronidazole), LMWH prophylaxis initiated
**Day 5**	Emergency laparotomy: entero-enteric fistula (10 mm), resection of 18 cm ileum, side-to-side anastomosis, retrieval of 13 magnets
**Postop 24 h**	CRP 120 → 75 mg/L; WBC 15.0 → 11.0 × 10^9^/L; continued IV antibiotics + LMWH
**Postop 48 h**	Oral clear liquids initiated; CRP 75 → 50 mg/L; WBC 11.0 → 9.5 × 10^9^/L
**Postop 72 h**	Transition to soft diet; CRP 50 → 25 mg/L; WBC 9.5 → 8.5 × 10^9^/L
**Postop Day 5**	Bowel transit restored
**Postop Day 7**	Discharged in good condition; antibiotics completed; LMWH stopped

**Table 3:** Comparative Management Approaches for Multiple Magnet Ingestion in Pediatric and Adult Populations.

Aspect	Pediatric Algorithm (NASPGHAN/ESPGHAN, Multicenter Data)	Adult Adaptation (Case Series, Reports)
**Case frequency**	Common; majority of magnet ingestion cases [6]	Rare; often intentional or psychiatric-related
**Primary goal**	Prevent perforations/fistulas; minimize surgery	Prevent complications; address psychiatric context
**Initial workup**	AP + lateral abdominal X-ray; US/CT if complications suspected [6]	AP + lateral X-ray; CT often used early for localization and complication assessment [29]
**Single magnet approach**	Observe ≤ 24 h; confirm progression [6]	Same as pediatric
**Multiple magnet approach**	Urgent endoscopy if accessible; otherwise prompt surgery [6]	Same principle, but lower surgical threshold if ingestion uncertain or patient noncompliant [29]
**Role of endoscopy**	Primary for magnets in stomach/duodenum	Primary if accessible; distal symptomatic cases often go directly to surgery
**Monitoring**	Inpatient, NPO, X-ray every 8–12 h	Same, plus routine psychiatric consult
**Particularities**	Standardized international algorithms	No dedicated adult guidelines; individualized adaptation

**Table 4:** Reported cases of magnet ingestion with or without fistula, stratified by age and fistula status.

Author	Year	No. of Magnets	Adult/Child	Age	Fistula Location	Fistula (Yes/No/Not Reported)	Treatment
**Özcan, R.** [6]	2024	Multiple	Child	6 years	Jejuno-colonic + volvulus	Yes	Surgical (fistula repair + devolvulation)
**Blevrakis, E.** [3]	2018	2	Child	9 years	Jejuno-jejunal (two loops)	Yes	Surgical (laparotomy)
**Doklestić, K.** [4]	2017	2	Adult	21 years	Jejuno-ileal	Yes	Surgical (primary suture of perforations)
**Arshad, M.** [11]	2019	Multiple	Child	2 years	NA	No	Surgical
**Zachos, K.** [12]	2019	Multiple	Child	4 years	Jejuno-ileal (double)	Yes	Surgical
**Phen, C.** [27]	2018	13	Child	19 months	Gastroduodenal	Yes	Endoscopic + supportive
**Pogorelić, Z.** [25]	2016	25	Child	2 years	Entero-enteric	Yes	Surgical (resection + anastomosis)
**Freeman, J.** [16]	2025	2	Child	10 years	NA	No	Endoscopic (colonoscopy, extraction)
**Goparaju, N.** [21]	2025	44	Child	2 years	NA	No	Multidisciplinary (ENT + endoscopic; remainder passed spontaneously)
**Sodagum, L.** [20]	2024	Multiple	Child	17 months	Small bowel loops (“seal ring” sign)	Yes	Surgical
**Balaswad, M.** [23]	2024	Multiple	Child	5 years	Entero-enteric + obstruction	Yes	Surgical (fistula excision + anastomoses)
**Ahmed, H.** [31]	2025	1	Adult	45 years	NA	No	Endoscopic (Roth net)
**Hakimzadeh, M.** [22]	2025	Multiple	Child	2 years	NA	No	Endoscopic

## Data Availability

The data presented in this study are available on request from the corresponding author. The data are not publicly available due to patient confidentiality.
